# Early Prediction and Evaluation of Risk of Autism Spectrum Disorders

**DOI:** 10.7759/cureus.23465

**Published:** 2022-03-24

**Authors:** Nada S Ashmawi, Manal A Hammoda

**Affiliations:** 1 Medicine and Surgery, Taibah University, College of Medicine, Madina, SAU; 2 Clinical Biochemistry, Taibah University, College of Medicine, Madina, SAU

**Keywords:** maternal factors, mmr vaccine, risk factors, autism, asd

## Abstract

Autism is a neuro-developmental condition that first appears at less than three years of age. Autism spectrum disorders (ASD) include several symptoms, such as social communication impairment, stereotyping behaviors, speech abnormalities, and impairment of eye contact. Its prevalence has increased recently, and several factors play a role in increasing the risk of autism. Multiple studies and research explain the factors affecting the rate of autism, and in this article, we will review most of these factors. The aim of this review article is to increase awareness of the problem of autism and provide scientifically relevant information about the etiology, pathogenesis, risk factors, and management of ASD. Our perception of autism has evolved over time. A few years ago, the condition was nothing more than an unrecognized developmental delay, generally with intellectual disabilities. Today, it is recognized as a major public health issue and a topic of much research. Researchers have struggled to find a cause for ASD, and numerous treatments have been developed to maximize the potential to learn and become socially fluent, no matter how strong the impairments may be. Although there is no cure, there have been significant strides in identifying and developing treatments. Early prediction of autism is beneficial in an individual's treatment, which can be carried out by recognizing the risk factors of infants, thus leading to improved outcomes or even a complete cure. The prevalence of ASD has increased, and earlier prediction leads to the best outcomes.

## Introduction and background

Autism spectrum disorder (ASD) is a lifelong disorder referred to as a group of neuro-developmental heterogeneous disorders sharing prevalent clinical features, including social-interaction impairment, language regression, repetitive or stereotypical behavior, narrow interests, and abnormal communication with others [1,2]. ASD is a broad term that involves three disorders: Asperger's syndrome (AS), autistic disorder (AD), and pervasive developmental disorder (PDD) [3]. The severity of features varies among children with autism ranging from mild to severe symptoms that may be associated with poor outcomes. However, the presence of genetic and environmental factors together may increase the severity of the disease. Autism is not generally suspected until the child reaches three years of age because parents usually do not notice early signs until the child starts to speak [4]. Characterized by a loss of non-verbal communication skills and only a few words, symptoms generally appear in early childhood, whereas for some patients, the symptoms may occur late in life [5].

There is an unclear increase in the prevalence of ASD, so it is described here as one of the modern medical challenges. One study estimated that there had been 42,500 confirmed instances of autism in 2002 in Saudi Arabia, and there may be other undiagnosed cases [6]. According to the Centers for Disease Control and Prevention (CDC) and the Autism Developmental Difficulties Monitoring Network (ADDMN), there was a 78% increase in ASD prevalence during 2002-2008 in the USA, with about one in 88 children identified with autism in March 2012. However, the autism burden increased, and in 2014 autism affected one in 68 children, with the male to female ratio at about 4:1 [5,7]. Another study in 2012 showed that the prevalence of parents having an autistic child has increased by 25% compared with the general population [8]. Since the 1960s, the unique relationship between autism and epilepsy has been a scientific topic for inquiry [7]. Despite global research, the etiology and pathogenesis of ASD remain unclear. It is supposed that a combination of de novo inherited variation and environmental factors may cause ASD. However, the lack of knowledge on ASD may lessen the opportunities for the treatment of autism [1,8,9].

Several conditions associated with ASD have been found, such as gastrointestinal abnormalities, toxins, drugs, and environmental factors [7]. Early work by Braam et al. was concerned with the fact that there is a strong association between genes and ASD pathogenesis: 800 genes have been discovered, and they were the cause of 25% of ASD cases. However, gene polymorphisms are not enough to explain the disease. In contrast, one study shows that genetic causes are responsible for only 1% of ASD cases [1,7,8]. Some studies have suggested that up to 60% of autistic patients have different types of immune dysfunction and many reports bind autism with immunological dysfunction. Both astroglia and microglia cells are involved in many central nervous system (CNS) disorders in pathogenic inflammatory mechanisms, and different factors might trigger them [2]. There is a direct impact of immune dysfunction in some or even all of these processes [10]. A study by Wing and Shah. stated that catatonia is considered to be a comorbid syndrome of ASD. It is present in 17% of the studied group aged 15 years and above, as shown in two systemic clinician-based studies [11].

Many studies have suggested that there is an association between brain lesions and behavioral outcomes; one study showed abnormalities in different regions of the brain that are all involved in the pathogenesis of autism: the frontoparietal cortex, hippocampus, amygdala, anterior cingulate cortex, basal ganglia, and cerebellum. Lesions in such areas prove the causal relationship between brain structure lesions and behavioral outcome difficulties [2]. Additional work by Tuchman et al. deals with autistic regression: one-third of children with autism develop social and language regression, termed 'autistic regression' because it occurs before the age of two years in most instances. There is a suggestion that autistic regression is accompanied by a higher rate of epilepsy, as one recent study found that autistic children with autistic regression had more disrupted sleep and a higher risk of epilepsy [2]. Some researchers found that there is a relationship between autism and epilepsy syndrome, reflected in the autism-epilepsy phenotype, which suggests that there are etiology and pathology associations between seizures and social, cognitive, and even communicative behaviors in autism. Both autism and epilepsy are heterogeneous disorders, although epilepsy syndrome is not one of the causal factors of autism [2].

Another study by Braam et al. found that melatonin is a crucial factor for normal embryological development as well as neurodevelopment; the embryo cannot produce melatonin, so maternal melatonin crosses the placenta barrier to reach the fetus, and its circadian level follows the maternal rhythm [8]. Several studies discuss pharmacological and non-pharmacological treatment options, using preclinical studies over the last two decades to identify the molecular pathways associated with cellular processes implicated in ASD and related conditions. Progress in pharmacological treatment targets has reached the clinical trial stage in several conditions related to ASD, such as Rett syndrome [5]. Evidence-based therapy is a behavioral analysis used to show that a patient's prognosis depends on early detection, diagnosis, and intervention of ASD, with several studies showing that significant benefits could result if intensive therapy is given during the first three years of life [9].

In summary, this review article aims to increase awareness of autism and provide scientifically relevant information about the etiology, pathogenesis, risk factors, and management of ASD.

## Review

Definition

ASD covers a group of neurodevelopmental disorders recognized by the early appearance of repetitive behaviors, either sensory or motor, and social communication abnormalities, associated with genetic and environmental factors. Unlike individuals 50 years ago, many patients with ASD today have mild symptoms and can read, speak and live with the general population in the community. Furthermore, a large number of individuals with ASD become symptom-free by adulthood [12].

There are apparent differences between this group of disorders regarding the etiology and symptomatology. Furthermore, the coexistence of intellectual disabilities with ASD is shown: about 40% of individuals with intellectual disabilities have ASD, whereas 70% of individuals with ASD have an intellectual disability. These frequencies may raise the question of whether such coexistence is due to both sharing similar causes. Additionally, the risk of ASD increased in more severe cases of intellectual disabilities. However, the risk of intellectual disabilities is greater in severe cases of ASD [8].

Epidemiology

In 2010, the CDC found that one out of 68 children in the USA has autism; the prevalence of ASD in males (1/42) is higher than in females (1/189), as shown by another study conducted by the CDC [4,5]. In addition, the worldwide prevalence of ASD is estimated by the World Health Organization (WHO) to be 1/160, considering 7.6 million disability-adjusted life-years. The WHO also estimated the global burden of ASD to be 0.3%, but in middle-income countries, the prevalence is still unclear [4].

Autism is a global disease, but few studies were conducted in the Middle East, with most of the research done in Western countries. In 2002 the prevalence of autism in the Kingdom of Saudi Arabia was slightly higher than in developing countries, and the number of confirmed cases was 42,500 [4,5].

Pathogenesis

The exact pathogenesis of ASD is still unclear, but several factors have been implicated. Recently, studies highlight several epigenetic phenomena such as skewed X-inactivation, gender-specific effects of Y-linked genes, parent-of-origin allelic genes, and escaping X-inactivation in the etiology of ASD and gene total expression, as well as the heterogeneity of gene regulation at the level of alleles [13,14]. Additional work by Samsam et al. confirmed that hormonal and genetic differences might be the cause of gender differences that could initiate at an early development stage due to different individual responses to environmental factors and their interaction such as the responses to infections, diet, stress, and drugs [7].

The formation of the placenta and its epigenetic process depends on many X-linked genes, which is why the placenta has an important role in gender-specific responses to environmental factors and in later stages of the disease. The etiology of ASD has long been affected by internal and external environmental factors. Activation of maternal immunity at an early stage may lead to prenatal stress, which influences boys more severely due to their defenseless genotype [7,13,14].

Risk factors

Genetic Predisposition

Al-Salehi et al. demonstrated that several factors were implicated to have a role in the pathogenesis of ASD, although the exact mechanism is still unknown. Genetic factors have a solid evidence-based etiology in autism [5,6]. Evidence found that genetic causes are the most prominent etiology in ASD individuals, and some diseases such as Rett syndrome and fragile X syndrome co-occur with ASD. Studies of twins indicated a strong role in inheritance. Other studies suggested that offspring of people with ASD have a higher incidence of inherited ASD than the general population. Several genetic studies discovered that abnormal structures in multiple scaffolding or transmembranous proteins were implicated in synaptogenesis, and the signal transduction mechanism of synapse formation involved in gene dysregulation is considered to be one of the fundamental gene abnormalities involved in ASD [7].

With the discovery of different genes as well as connections of these several genes in a single individual, epigenetic factors, and impacts of ecological modifiers on these qualities in ASD, hereditary causes, including the diagnosable ailments, single-quality imperfection, and cytogenetic issues, contain 25% of the ASD patients so far. Consequently, ASD is characterized by several clinical phenotypes and associated comorbidities. Some studies suggested that mutations in DNA mitochondria may have a role in ASD, affecting mitochondrial energy metabolism. However, further studies are needed for a definitive answer [7]. The role of mitochondria in antibacterial immunity is significant in infections, particularly in gastrointestinal tract (GIT) infections in a child with ASD [13]. The male to female ratio for ASD is 4:1; the reason for this is not well known, but it is significant [7].

Maternal Factors

Parental age: A study by Gardener et al. reported that advanced parental age, especially paternal age, is considered one of the most significant factors associated with ASD [15]. In several studies, a parental age greater than or equal to 34 years increases the risk of having autistic offspring. In contrast, other studies show no relation between parental age and autism.

Intriguingly, most studies agreed that there is a relationship between advanced paternal age and increased risk of autism [16,17]. Based on research conducted in 2010 among the Iranian population, there is a 29% increase in autism risk for every 10-year advance in the father's age. In other words, the autism risk increases by approximately twofold in the child of a father aged 34-39 years and by 2.58 for a father aged over 40 years compared to a father aged 25-29 years. Studies conducted in China and Japan show that the relationship between paternal age and increased autism risk is similar to the previous survey. In these three studies, the lack of any correlation between maternal age and autism susceptibility is critically significant. It can be explained by germline cell mutations and alteration in the methylation of DNA, which may lead to general epigenetic modifications in neural development gene expression and eventually genomic imprinting sperm disorders [18-20].

Maternal physical health: Other factors that may increase the risk of autism are metabolic syndrome, physical disease of the mother, infection, and bleeding during pregnancy [21]. There is a strong relationship between maternal bleeding during pregnancy and the risk of autism; the risk is elevated up to 81%. In addition, hypoxia in utero is associated with brain development deficiency, changes in the myelination process, hippocampal deficiency (an area in the brain strongly associated with autism), and membrane adhesion [15,22-24]. Hypoxia also results from metabolic syndrome (e.g., hypertension, hyperglycemia, and obesity) [20].

An additional survey by Karimi et al. discovered that the relationship between abnormal activation of a pregnant woman's immune system and high levels of inflammatory cytokines has a negative effect on embryonic brain development, consequently increasing the risk of autism and other neurological, pathological, and physiological conditions [20]. First-trimester maternal viral infections (e.g., herpes, rubella, measles, Borna disease virus, influenza, chickenpox, varicella-zoster, pneumonia, mumps, syphilis, cytomegalovirus) [21,25,26], and second-trimester maternal bacterial infections that need hospitalization will cause offspring behavior almost identical to autism symptoms, such as social interaction and communication abnormalities, as well as repetitive motor behavior [1]. These infections also increase the risk of autism in the fetus [25].

Maternal mental health: Parental behaviors, family identity, and communication patterns between parents all influence the development of children's personalities and emotional states [27]. There is an obvious correlation between a parent's psychiatric history and a child's mental disorders, particularly autism. For example, for parents with schizophrenia, the risk of having a child with autism increases threefold [16]. The susceptibility of autism increases with the mother's anxiety, depression, or other personality disorders, as proven in several studies [28-30]. Maternal mental illness between 21 and 32 weeks of pregnancy, a period of increased plasticity for the formation and development of the fetus, can have irreparable effects on the stress response gene of the embryo, the epigenetic mechanism, and genes associated with neurobiology, metabolism, and physiology that can be present over the lifespan [20,30].

Furthermore, the fetus is affected by the elevation of cortisol due to interruption of the mother's hypothalamic-pituitary-adrenal (HPA) axis in response to an unstable psychological state, particularly long-term stress, which may result in the mother's aggression. The placental permeability to hormones amplified by the adrenal cortex also increases stress hormones [20].

Vitamin D level: Other risk factors associated with ASD are dietary factors, which generally have been neglected until recently. The level of nutritional elements, which can also be affected by physical (sunlight) or pharmacological routes (supplements), has taken on new importance in studies on the relation between the environment and ASD. Susceptibility of ASD occurrence increases with vitamin D deficiency, either maternal during pregnancy or in the early years of her offspring. Studies suggest that low maternal vitamin D levels have a significant role in autism risk [31- 33]. Few studies found low maternal vitamin D levels in mothers of children with autism compared to mothers of non-autistic children. Vitamin D plays a significant role in the process of repairing DNA and also has anti-inflammatory effects on brain tissue and other biological processes [34]. 

Folic acid: The risk of autism is affected by the availability of folic acid. Folate plays a significant role in fetal neurodevelopment, and it is thought that the folate level affects the metabolic pathway of methionine. Deficiency in folic acid negatively affects metabolic pathways and is thus considered a causative factor of autism [17]. Dietert et al. discovered that the two possible factors influencing metabolic pathways are autoimmune folic acid receptors and pathway genetic polymorphism [34]. As stated in a recent review of this subject [35], for definitive conclusions, the heterogeneity of studies on folate and autism to date poses a problem. The ability to examine potential interactions between folate levels, genetic background, and gene expression is particularly varied in these studies [34,35].

Melatonin level: One of the most significant risk factors of ASD is a low level of melatonin, which increases susceptibility to ASD environmental risk factors. The fetus is unable to secrete melatonin, and therefore a low maternal melatonin level increases the risk of ASD. It has been shown that the pineal gland is responsible for melatonin synthesis in the dark phase during the day. It has multiple functions and plays a role in inducing sleep and regulation of the circadian rhythm. Melatonin can also reduce oxidative stress effects and act as a free radical scavenger and antioxidant; organ injury induced by pesticides can be prevented or at least minimized by melatonin because of its antioxidant effects [8,15,21]. There is also evidence that melatonin protects against DNA damage caused by oxidative stress.

The majority of autistic individuals have a low level of melatonin, with the severity of autism increasing at lower melatonin levels. Acetylserotonin O-methyltransferase (ASMT) is proved to be one of the melatonin synthesis pathway factors, and it is suspected to be involved in ASD pathogenesis. In contrast, several studies show that ASMT does not affect ASD in the same way as it affects the healthy control group. Based on the genetic basis of ASD, no more than 1% of autism cases have chromosomal abnormalities. Thus, a low melatonin level is present in the majority of individuals with ASD, but it is considered comorbidity [8].

Perinatal Factors

Human developmental neurotoxicity is strongly associated with ASD and developmental delay if a mother is exposed to agricultural pesticides during pregnancy [7]. More than 200 identified chemicals have a neurotoxic impact on the brain and increase the risk of teratogenicity. One of the most studied factors is valproic acid (VPA). Clinically, VPA is used as an anti-epileptic and sometimes for psychiatric problems such as bipolar disorder. Using VPA in early pregnancy might increase the risk of autism threefold. The reason for VPA's teratogenic effects is that VPA increases oxidative stress production and inhibits histone deacetylase 1 (HDAC1) by binding to its active sites in the fetus's brain, which leads to autistic behavior. Competitive binding of VPA to the HDAC1 active sites induces histone deacetylase 2 (HDAC2) proteasomal degradation. HDAC1 plays a significant role in neurogenesis and gliogenesis and is expressed in neural/glial stem cells, whereas HDAC2 is expressed in postmitotic neuroblasts but not in glial cells that are fully differentiated. Using VPA may lead to different outcomes associated with ASD-like symptoms due to its effect on the modulation of HDAC in various types of cells and at several times [1].

Environmental Factors

A study by Dietert et al. confirmed that environmental factors could increase the risk of autism. Data are derived from both human and animal studies and experiments in the search for autism's ecological risk factors. Direct exposure of humans to ecological factors includes environmental chemicals, drugs, medical procedures, and maternal stress a short time before conception [34].

Stress: These are closely linked to the risk of autism, as some neurological researchers assume that the brain of autistic children may be a 'hyper-male' form and that fetal testosterone levels play a significant role in autism risk. In contrast, Li et al. did not agree with this relationship between stress and autism risk; they found no evidence of autism risk increasing after maternal exposure to prenatal stress. Fetal health and development are affected by several factors, such as the mental, physical and psychological health of the mother and even the financial state throughout the pregnancy. A mother who is mentally and physically unhealthy might not have a healthy neonate [20,34,36,37].

Drugs: It is reported in a previous study by Gardener et al. that a 46% increase in the risk of autism in the fetus is associated with maternal use of medication during the prenatal period. The risk of autism increases significantly up to 68% in children with maternal prenatal use of anti-psychiatric medications, as shown in several types of research [20]. Based on several studies, medications used in the prenatal period have a detrimental effect on the development of a fetus because they can easily cross the placenta: for example, anti-epileptic medications, such as VPA, which leads to 'fetal valproate syndrome', as well as it can lead to disturbances of normal growth manifesting by abnormal social behaviors, communication, and motor activity. The underlying mechanism is increasing the oxidative stress and abnormalities of gene expression​​​​​** **[21]**. **Furthermore, studies have proven that acetaminophen (paracetamol) is used as a pain reliever, and the antipyretic drug is responsible for causing brain structure changes, such as in autistic children having apoptosis and necrosis in brain tissue. Moreover, acetaminophen can promote oxidative stress production and dysregulation of immunity. On the other hand, many studies have not demonstrated a relationship between antidepressant medications and ASD.

Relationships with other drugs have been specified. These drugs include painkillers, thalidomide, misoprostol, and prostaglandin analog(used for peptic ulceration) in the first trimester of pregnancy, and also β2-adrenergic agonists used by asthmatic patients [20].

Studies showed a significant increase in environmental concerns related to increasing the risk of autism several-fold in the case of pharmacological drugs, especially during pregnancy. The prevalence of autism changes according to the availability of certain prescribed or over-the-counter medications [34,38]. In addition, maternal exposure to a small amount of distinct during pregnancy, such as VPA (an anti-epilepsy drug and stabilizer of mood) or thalidomide, increases the autism risk due to these drugs being considered as good examples of human teratogenic drugs. The risk extent depends on the dose of the drug and time of exposure [34]. 'Fetal valproate syndrome' is a well-known syndrome affecting multiple systems in children if their mothers take VPA during pregnancy because it can increase oxidative stress and affect gene expression [39]. The effects of VPA, including pre- and postnatal delay of normal development and deficits in social behaviors and motor activities, are well known. In humans, the condition is associated with changes in post-synaptic cell adhesion molecules [34].

Terbutaline is a β2-adrenergic agonist used in asthma. One study group showed that prenatal exposure to β2-adrenergic agonists is associated with an elevated risk of autism, along with other neurophysiological results, but this association is limited to human reports [34,40]. Another study stated that thalidomide's mechanism of action is by binding to cereblon protein and inhibiting the activity of ubiquitin ligase. As reported in two studies, prenatal exposure to thalidomide is associated with an increase in the risk of autism [34].

Heavy metals: There is veiled evidence that exposure to heavy metals can lead to an autistic phenotype. The most common reported metals strongly associated with the risk of autism are sulfhydryl-reactive metals such as arsenic, lead, cadmium, and mercury [34].

An additional study by Adams et al., in an experiment with autistic and control groups, found that the level of sulfhydryl-reactive metals was lower in hair samples of the autistic group compared with the control group. Due to poor excretion and detoxification of metals, this suggests that the autistic group increases susceptibility to certain metals, and other metals may increase the risk [41]. The severity of autistic symptoms was reported to be influenced by aluminum, antimony, lead, and mercury body burdens, as measured by urinary excretion. Further research on exposure to heavy metals and the risk of autism is needed, not only to identify the most common associated metals with increased risk but also to determine the most suitable risk assessment biomarkers [20].

Postnatal Risk Factors

Measles, mumps, and rubella (MMR) vaccine: A retracted paper of Wakefield et al. [42] mentioned that there were eight children showing autistic symptoms within one month after receiving the MMR vaccine; all of these children had nodular lymphoid hyperplasia and gastrointestinal signs, as seen by endoscopy. Wakefield et al. assumed that MMR vaccine is associated with abnormal brain development because it causes intestinal inflammation, which leads to the passing of non-permeable peptides to blood and then to the brain. Autism appears in the first 1-2 years of life, at the time of receiving the MMR vaccine. In England, about 25 children were diagnosed with autism shortly after receiving the MMR vaccine. The MMR vaccine has not been associated with chronic inflammation of the intestines or effects on barrier function. They found that the virus genome of the MMR vaccine was not commonly observed in autistic and non-autistic children.

Encephalopathic peptides have not been identified in the brain. On the other hand, genes associated with ASD have been found to code for intrinsic proteins influencing neuronal synapse function, adhesion of neuronal cells, endosomal trafficking, and neuronal activity regulation [43].

Researchers in the UK assessed 498 children with autism born between 1979 and 1992 who were located by computerized records from eight health centers; however, they did not find any change in the autism rate after the 1987 introduction of the MMR vaccine. Furthermore, autistic children's MMR vaccine rates were similar to those of the entire population of the study. In a study done in the UK, 71 autistic children who received the MMR vaccine and a control group of 284 non-autistic children who also received the MMR vaccine were compared using the Doctor's Independent Network database in general practice. Within six months of the MMR vaccine, the autism diagnosis could not be related to the vaccination: the authors observed no difference between case and control groups in the practitioner consultation rates - a measure of parental concern over the development of their child. In conclusion, 12 epidemiological studies performed by different investigators in different countries showed that the MMR vaccine does not cause autism [43].

Gastrointestinal problems: Gastrointestinal manifestations are commonly seen in ASD children as gastro-oesophageal reflux, vomiting, constipation, or chronic diarrhea. Immune system disturbance is usually seen in ASD and is directly associated with GIT problems. The period of the early development of organs can be affected by immunity activation and result in abnormal development [7,31]. GIT problems such as constipation, gastro-oesophageal reflux disease, vomiting, abdominal pain, and chronic diarrhea are highly indicated in many studies. In contrast, a case-control study based on a UK database suspected that there is no significant relation between gastrointestinal abnormalities and autism. Due to studies' eliminations and speculations, the role of increased intestinal permeability due to leaky gut or inflammation in ASD pathology has not been established [7,20].

Other studies may likely show that leaky gut and epithelial damage and leaking of dietary sources gluten or other products into the blood via injured intestinal barrier has no role in the brain's immunogenic responses. Mitochondria have an antibacterial effect on immunity; therefore, ASD individuals with immune system disturbance are more prone to infections, particularly in the GIT [7,20]. Studies have reported a strong association between the presence of GIT problems and ASD severity; individuals with severe ASD will suffer from severe disability later in life, and only 15% of autistic children will have a favorable life in adulthood [7].

Epilepsy: It is a paroxysmal disorder affecting brain activity and thus is associated with a wide range of abnormal behavioral manifestations. Seizures can be categorized into different syndromes according to the semiology of symptoms and convulsion characters, motor signs and symptoms, and electroencephalogram (EEG)-specific patterns. EEG seizure patterns have no clinically abnormal manifestations in cognition, behavior, or motor activity, so these patterns are subclinical or non-convulsive seizure patterns. Spike or spike changes in EEG or any other wave discharge on EEG are seen in epileptiform patterns. Autism and epilepsy disorders are strongly related to each other, and autism epilepsy has no single etiology. Social interaction disturbance is considered the most distinctive sign for autism over other developmental disorders. Autism and epilepsy are both common disorders, with prevalences of 5.8/1000 for autism and 7.1/1000 for epilepsy. Studies estimated that the prevalence of children with autism having epilepsy, in general, is 30%, as seen in a tertiary clinic of epilepsy. Children having both disorders at the same time have worse developmental trajectories than children having just one of these disorders. The most prevalent risk factor recorded in studies on the development of epilepsy disorder in children with autism is moderate to severe cognitive impairment or mental retardation. Epilepsy prevalence in children with autism and mental retardation is as high as 40%. Studies have estimated that autistic children with mental retardation have the highest rate of epilepsy. The prevalence of epilepsy in a child with autism who is not mentally affected is only 1%, whereas the prevalence in those having other disorders with cognitive impairment is markedly higher; in children with Down syndrome or cerebral palsy, the prevalence is 13%, but this markedly increases in those with both cerebral palsy and cognitive impairment. Moreover, in the primary group, severely mentally affected individuals have a higher rate of seizures (20%) than moderately affected individuals (12%), showing that the severity of brain affection and mental retardation directly affects the appearance of epilepsy. The rate of epilepsy in autistic children with no family history of seizures or any risk factors and no significant motor affection or severe mental retardation is about 6%, which is similar to the rate of the control group without autism but with language impairment [2,13,16].

The risk of poor outcomes in children having seizure onset before age five years is the highest, as it is strongly associated with low functional intelligence. In one study of 246 autistic children, 6.5% with seizures, it is shown that 80% started their seizures in the first year of life. Seizures may modify gene expression and the release of neurotransmitters, leading to changes in EEG patterns that affect the cognitive state of epileptic or autistic children. The development of gamma-aminobutyric acid (GABA)ergic interneurons depends on several genes that may be present in both autism and epilepsy. GABA plays an important role in brain development, particularly in autism and epilepsy, but it is a complex process [13,16].

Other Postnatal Risk Factors: Lesser

Several postnatal manifestations such as jaundice, infection, and low birth weight are significantly associated with a higher risk of autism. Three potential factors may result in low birth weight: genetic factors, pregnancy duration, and fetal growth rate [16]. Birth weight less than 2.5 kg increases the risk of autism twofold [44]. A study by Zhang et al. [18] confirmed that the general immaturity of the fetal liver affects its capacity to excrete bilirubin (which is produced from a high rate of erythrocyte breakdown) so that postnatal jaundice appears, which is strongly associated with death during the 40 weeks of pregnancy, which is a sensitive period, or increased vulnerability to cognitive impairment. If the fetus survives, the risk of autism is increased fourfold [16,18]. The risk of autism is also increased in postnatal (and prenatal) infections such as meningitis [26], varicella, mumps, unknown fever, and infection of the ear in the first month of life [20]. One of the most studied theories in ASD is immune system alteration, either innate or acquired. Data focusing on the role of immune disturbance is still lacking, despite research efforts. A study of 31 patients with ASD and several immune disturbances was published in this field in the 1980s, with ASD patients showing a high level of pro-inflammatory cytokines in their blood [7,22].

Elevated leukocytes of T helper (Th) 2-associated cytokine production are seen in patients with autism. However, in children with autism, there is no strong evidence of changes in the total number of blood T cells. Instead, there is a clear disparity in T cells in patients with ASD, as well as natural killer cells. As implicated in several studies, immune system abnormality supports the immunity hypothesis involved in ASD pathogenesis. For example, microglia are found in the cerebrospinal fluid of ASD individuals. Recently, neuroimmune abnormalities have been examined. The blood-brain barrier (BBB) is a major brain homeostasis regulator, but neurological inflammation, increased inflammatory cytokines in the brain, and immune system abnormality may alter the function of the BBB in children with ASD. Abnormal immune responses are observed in the GIT, CNS, and peripheral blood of ASD patients [7,22]. Studies have observed a significant decrease in two types of lymphocytes, CD4 and CD8, similar to the inequality between T helper (Th) 1- and Th2-like cytokines in autism. Also, an imbalance between interferon-gamma (IFN-γ) and interleukins and the overactivity of the pathways of Th1 and Th2 has been implicated in the peripheral blood of ASD children. An imbalance of immunoglobulins was reported in ASD children. Total protein of ASD children's serum was significantly changed, and there was an increase of serum albumin and γ-globulin levels, as well as immunoglobulin G2 (IgG2) and IgG4, which possibly contributes to autoimmune disorders and thus increases the probability of infections [7].

In general, recent data approved that there is dysregulation of ASD patients' immune systems. Interestingly, as shown in several studies, intravenous injection of IgG to patients with autism could improve symptoms such as speech abnormalities, eye contact, and quiet behavior, and also temporarily may improve the attention and hyperactivity reported [1].

Familial Socioeconomic Status

In general, autistic children and their families have poor economic, psychological, educational, and social aspects. Fundamentally, they experience psychological and occupational stress, social instability, and due to financial problems, have unresolved life events [20,38]. Inability to access proper healthcare or enjoyment facilities emerges as physical health impairment and infection [45]. In addition, the experience of stress and anxiety (e.g., from living with other families) causes psychological tension in parents, especially mothers during pregnancy, and increases the susceptibility of having a child with autism [18]. It was reported in a previous study by Karimi et al. that the psychological state of a pregnant woman is strongly affected by their isolation and reduced social interaction and communication, which not only affects the mother's health but also that of the embryo [20]. The relationship between a low level of parental education and autism is discussed in several studies, with the results showing that there is a relation between the two; in contrast, other studies suggest a relation between high education level and autism [38,46].

Pharmacological treatment

Antipsychotics

The United States Food and Drug Administration (FDA) has approved only two drugs to be used in ASD: risperidone and aripiprazole. The mechanism of action of these two drugs remains unknown. The target of these medications is not ASD itself but its behavioral symptoms. It was suggested that risperidone's therapeutic action could be mediated by a combination of type 2 dopamine (D2) and type 2 serotonin (5HT2) receptor antagonism. In contrast, aripiprazole's therapeutic effect is achieved via a combination of incomplete agonist action on both D2 and 5HT1A receptors and antagonist action on 5-HT2A receptors. Various studies have stated that because these two drugs target behavioral abnormalities in ASD individuals, the indications include irritability and disruptive behaviors as well as self-injury behaviors that result from ASD. These two medications showed great results in reducing hyperactivity and stereotyped behaviors. However, risperidone and aripiprazole have serious side effects, such as dysregulation of plasma lipid, increased blood glucose, and weight gain (Table 1). A recent systematic study showed that N-acetylcysteine significantly improved some ASD symptoms, such as irritability. In a small randomized controlled trial (RCT) study, the active drug group showed greater improvement of ASD symptoms in comparison with a placebo group; however, the study participants also took other medicines, as well as behavioral interventions, throughout the study. An additional study by Ghanizadeh and Moghimi-Sarani was not able to show that N-acetylcysteine in conjunction with risperidone improved ASD core symptoms, despite finding some improvement in irritability for ASD individuals (Table 1) [5, 47].

**Table 1:** Pharmacological treatment of autism spectrum disorders [5] ASD - autism spectrum disorders

Treatment	Disorder	Implicated gene or pathway	Suggested pathological mechanism	Suggested treatment mechanism	Outcomes
Risperidone (FDA approved)	ASD	Dopamine and serotonin signaling	Dysregulation of dopamine and serotonin receptor activity	Combination of dopamine type 2 (D2) and serotonin type 2 (5HT2) receptor antagonism	Reduced irritability, stereotypy and hyperactivity but not effective at improving communication or social deficits
Aripiprazole (FDA approved)	ASD	Dopamine and serotonin signaling	Dysregulation of dopamine and serotonin receptor activity	Combination of partial agonist activity at dopamine type 2 (D2) and serotonin type 1A (5-HT1A) receptors and antagonist activity at 5-HT2A receptors	Reduced irritability, stereotypy and hyperactivity, but not effective at improving communication or social deficits
Oxytocin (under clinical trial)	ASD	Oxytocin signaling	Disrupted expression of CNTNAP2, C581J, GRIN1	Modulates auditory-visual attention and social sensory processing; regulates signal to noise ratio; restores gamma-aminobutyric acid (GABA) from excitatory to inhibitory neurotransmission	Improved social reciprocity, facial emotion, recognition, facial mimicry, eye gaze, trust and emotion perception and processing

Oxytocin

Insel and Young confirmed that oxytocin is a neuropeptide formed in the hypothalamus that plays an important role in social attachment and interaction in mammals. Several trials in healthy adults found great improvement in social behaviors such as social mimicry, emotion perception, eye gaze, and trust when intranasal oxytocin was used [5,48].

Non-pharmacological Treatment

An RCT study was performed, and the results showed a greater improvement in autistic individuals who received neurofeedback treatment; this improvement includes social interaction and communication skills. Neurofeedback is more beneficial than other treatments because it has a shorter treatment time and gives long-term improvement. There are two types: deep brain stimulation (DBS) and transcranial magnetic stimulation (TMS). In TMS, a simple coil is used to produce a magnetic field on the head, which in turn stimulates the brain area. It is typically effective and well-tolerated, with few adverse effects. DBS is the surgical procedure of implanting electrodes in specific areas of the brain. Studies have proved that it has therapeutic benefits, and it is considered as an alternative treatment for individuals resistant to pharmacological options or for those with comorbidities and low quality of life. Sturm et al. found that DBS of brain areas such as the basolateral amygdala and globus pallidus internus in autistic individuals result in significant improvement of social and cognitive symptoms and a reduction of stereotypic and self-injurious behaviors [5,49].

## Conclusions

In summary, ASD's specific pathogenesis is unknown and, as a result, is associated with different risk factors that might increase the prevalence of occurrence. These risk factors include genetic predisposition, maternal and perinatal-related factors, as well as environmental factors. Possible treatment options are pharmacological and non-pharmacological and are widely available. However, a complete cure for ASD is not found yet. In general, we would attract the attention of readers and general populations to increase the awareness about ASD as it is not an uncommon condition worldwide and much more of its risk factors are preventable, and early prediction will lead to better prognosis and lifelong outcomes.
